# The Lyme Disease Pathogen Borrelia burgdorferi Infects Murine Bone and Induces Trabecular Bone Loss

**DOI:** 10.1128/IAI.00781-16

**Published:** 2017-01-26

**Authors:** Tian Tian Tang, Lucia Zhang, Anil Bansal, Marc Grynpas, Tara J. Moriarty

**Affiliations:** a Matrix Dynamics Group, Faculty of Dentistry, University of Toronto, Toronto, ON, Canada; b Department of Pharmacology and Toxicology, University of Toronto, Toronto, ON, Canada; c Lunenfeld-Tanenbaum Research Institute of Mount Sinai Hospital, Toronto, ON, Canada; d Department of Laboratory Medicine and Pathobiology, University of Toronto, Toronto, ON, Canada; Washington State University

**Keywords:** Borrelia burgdorferi, Lyme disease, bone, bone mineral density, histomorphometry, host-pathogen interactions, infectious disease, microcomputed tomography, mouse model, osteopenia

## Abstract

Lyme disease is caused by members of the Borrelia burgdorferi sensu lato species complex. Arthritis is a well-known late-stage pathology of Lyme disease, but the effects of B. burgdorferi infection on bone at sites other than articular surfaces are largely unknown. In this study, we investigated whether B. burgdorferi infection affects bone health in mice. In mice inoculated with B. burgdorferi or vehicle (mock infection), we measured the presence of B. burgdorferi DNA in bones, bone mineral density (BMD), bone formation rates, biomechanical properties, cellular composition, and two- and three-dimensional features of bone microarchitecture. B. burgdorferi DNA was detected in bone. In the long bones, increasing B. burgdorferi DNA copy number correlated with reductions in areal and trabecular volumetric BMDs. Trabecular regions of femora exhibited significant, copy number-correlated microarchitectural disruption, but BMD, microarchitectural, and biomechanical properties of cortical bone were not affected. Bone loss in tibiae was not due to increased osteoclast numbers or bone-resorbing surface area, but it was associated with reduced osteoblast numbers, implying that bone loss in long bones was due to impaired bone building. Osteoid-producing and mineralization activities of existing osteoblasts were unaffected by infection. Therefore, deterioration of trabecular bone was not dependent on inhibition of osteoblast function but was more likely caused by blockade of osteoblastogenesis, reduced osteoblast survival, and/or induction of osteoblast death. Together, these data represent the first evidence that B. burgdorferi infection induces bone loss in mice and suggest that this phenotype results from inhibition of bone building rather than increased bone resorption.

## INTRODUCTION

Lyme disease is the most common vector-borne disease in the Northern Hemisphere (1). Its causative agents are spirochetes from the Borrelia burgdorferi sensu lato species complex (2). These bacteria are not known to secrete conventional toxins and are therefore postulated to induce pathology primarily by triggering host inflammatory response (3). B. burgdorferi is transmitted to vertebrates during the blood meal of Ixodes spp. ticks and disseminates from the tick bite site to colonize a variety of host tissues. This can result in diverse clinical manifestations at various infection stages (4). In humans, erythema migrans (EM), a bull's-eye-shaped skin lesion, typically appears at the bite site 1 to 2 weeks postfeeding. If antibiotic treatment does not begin at this early infection stage, B. burgdorferi disseminates to more distant tissues, and late-stage manifestations such as Lyme arthritis, carditis, and neuroborreliosis can develop. Of these late-stage symptoms, Lyme arthritis is the most common in North America, affecting as many as 60% of untreated patients (5). The pathological effects of B. burgdorferi infection on bone tissue outside articular surfaces, however, have not been widely investigated.

Bone pathologies are observed in diseases associated with other spirochete bacteria, including syphilis and periodontitis (6, 7). Bone is central to physiological homeostasis of the whole body and to systemic responses to infection, injury, and a variety of stressors (8). Infections within bone, as well as induction of local and systemic inflammatory responses to infection at other sites, can be accompanied by significant bone loss (8, 9). B. burgdorferi has been detected by culture and PCR in bone and marrow of humans, dogs, and the mummy of a Copper Age man (10–13). Furthermore, migratory bone pain, bone erosion at articular surfaces, multifocal osteomyelitis, and other bone involvement have been reported in B. burgdorferi-infected rodents and humans (5, 12–21). In the course of other studies requiring collection of bone marrow, we have noticed that long bones from B. burgdorferi-infected mice occasionally appear to be more brittle than bones of mock-infected controls. This prompted us to examine whether B. burgdorferi induces structural and cellular pathology in murine bones.

## RESULTS

### B. burgdorferi infection reduces serum alkaline phosphatase activity, a biomarker of bone formation.

Alkaline phosphatase (ALP) catalyzes monoester hydrolysis and is involved in many physiological processes, including bone formation (22, 23). The liver and bone-building osteoblasts are two examples of numerous sources of ALP in the body (24). Unlike in humans, where serum ALP is derived primarily from the liver, in mice serum ALP consists mainly of tissue-nonspecific bone isotype ALP (bone TNAP) and therefore serves as a biomarker of bone formation (24, 25).

**FIG 1:**
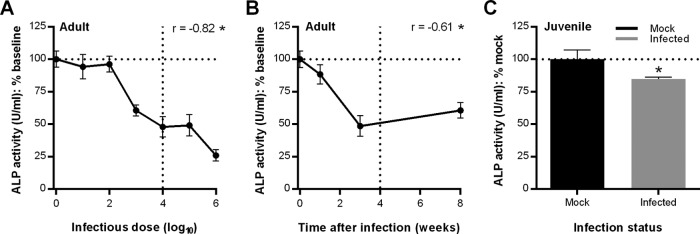
Effect of B. burgdorferi infection on ALP, a serum marker of bone formation. The mean (± standard error of the mean) serum ALP activity is shown, after normalization to the mean baseline ALP activity immediately preceding infection (0 weeks) (A and B) or in mock-infected mice (C). Primary data are presented in Fig. S1 in the supplemental material. (A and B) Serum ALP activity in adult (16- to 17-week-old) male C3H/HeN mice infected for 3 weeks with 10^1^ to 10^6^ B31-derived GCB726 B. burgdorferi bacteria (A) or for 1, 3, and 8 weeks with 10^4^ bacteria (B). *n* = 5 mice/group (A) or 10 mice/group (B). Statistics used were Pearson correlation analysis (*r*). *, *P* < 0.05 versus dose (A) or time (B). (C) Serum ALP activity in juvenile (3- to 4-week-old) male C3H/HeN mice infected for 4 weeks with 10^4^ B. burgdorferi cells. Mock is the age-matched mice inoculated with vehicle alone. *n* = 10 mice/group. Statistical analysis used a two-tailed unpaired *t* test. *, *P* < 0.05 versus mock-infected controls.

In 16-week-old adult male C3H/HeN mice infected with 10 to 10^6^ B31-derived B. burgdorferi for 3 weeks, serum ALP activity declined with increasing infectious dose relative to ALP levels immediately before infection, especially for mice inoculated with ≥10^3^ bacteria (Fig. 1A). In mice infected with 10^4^ B. burgdorferi, serum ALP activity declined with increasing infection duration, with the most prominent reduction in activity observed at 3 weeks postinoculation (Fig. 1B), when pathological outcomes of B. burgdorferi infection are typically most pronounced in C3H mice (26). Juvenile mice, which are typically more commonly used for Lyme disease studies (27), also displayed reduced serum ALP activity after 4 weeks of infection with 10^4^ B. burgdorferi bacteria compared to mock-infected mice (Fig. 1C). The effect of B. burgdorferi infection on serum ALP activity was less prominent than in older mice, likely because bones in juvenile animals are still undergoing rapid bone formation and skeletal development (28). These data prompted us to investigate whether B. burgdorferi colonizes bone and/or affects bone health.

### Bone colonization by B. burgdorferi.

We examined B. burgdorferi colonization and infection effects on bone in 12-week-old adult male C3H/HeN mice. At this age, murine bone development stabilizes, and peak bone mineral density (BMD), trabecular volume, and cortical thickness are attained (28, 29). Mice were infected for 4 weeks with 1 × 10^4^ B31-derived B. burgdorferi bacteria. Bone morphology, cellular composition, mineralization, growth rate, and biomechanical properties were investigated in long bones (tibiae, femora) and vertebrae, since long bones and vertebrae can exhibit different degrees of response to external stimuli. For example, rosiglitazone, a common antidiabetic drug, increases fracture risks in women in the limbs but not in vertebrae (30).

**FIG 2:**
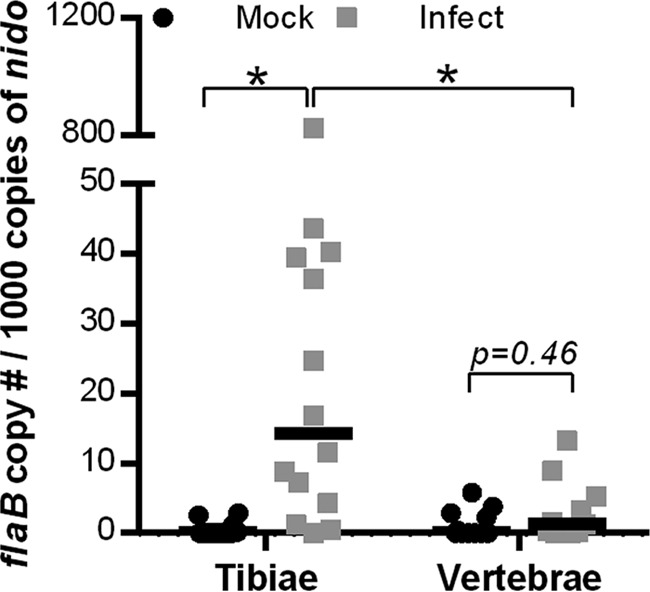
Detection of B. burgdorferi DNA in bones. Quantitative real-time PCR measurement of copies of the B. burgdorferi flaB DNA sequence in distal tibial halves and L6 vertebrae from 12-week-old male C3H/HeN mice 4 weeks after inoculation with 10^4^ B. burgdorferi cells or vehicle alone (mock). Shown are median and individual numbers of *flaB* copies per 1,000 copies of the mouse nidogen gene (*nido*) in each sample. *n* = 14 to 15 per group. Statistical analysis used a two-way analysis of variance with the Holm-Sidak posttest of log-transformed values. *, *P* < 0.05.

We first investigated whether B. burgdorferi could colonize murine bone. DNA was extracted from cleaned, crushed L6 vertebrae and distal halves of tibiae from which associated soft tissues and bone marrow had been removed (see Materials and Methods). Distal regions of tibiae were examined to ensure samples were collected as far from joints as possible, since joints are typically readily colonized by B. burgdorferi. It was more difficult to remove all surrounding soft tissues from vertebrae than from tibiae. Although we were confident that any bacterial DNA detected in tibiae likely originated from bone itself, we could not be certain that B. burgdorferi DNA detected in vertebrae was entirely bone derived. Total copies of a portion of the B. burgdorferi flaB gene and mouse nidogen DNA sequence were measured in each sample by quantitative real-time PCR (qPCR), and the *flaB* copy number/1,000 copies of nidogen was compared in different bones of mock-infected and infected mice (Fig. 2). *flaB* DNA copy number was significantly greater than background in mock-infected animals in the tibiae but not in vertebrae of infected mice (Fig. 2), indicating that tibiae were colonized more effectively. The *flaB:nidogen* copy number ratios in tibiae were similar to those previously reported in other tissues of B. burgdorferi-infected mice (31), implying that B. burgdorferi can colonize regions of long bones distal to articular surfaces as efficiently as other tissues.

### B. burgdorferi infection induces osteopenia in the trabecular region of long bones.

**FIG 3:**
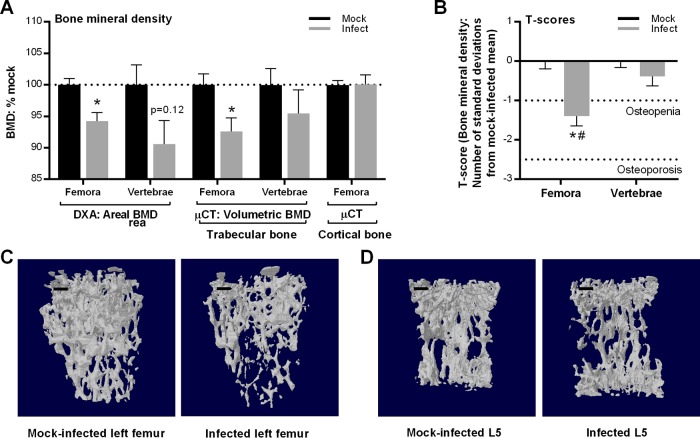
Effect of B. burgdorferi infection on bone mineral density. Areal and volumetric bone mineral densities were measured by DXA and μCT, respectively, in distal femoral metaphyses and L5 vertebrae from 12-week-old C3H/HeN male mice infected for 4 weeks with 10^4^ B. burgdorferi cells or mock infected with vehicle alone. Values were normalized to mean values for mock-infected mice within each bone type and measurement method group. Primary data are provided in Table S1 in the supplemental material. (A and B) Mean (± standard error of the mean) areal and volumetric BMDs (A) and BMD T-scores (B). (C and D) Representative 3D μCT models of trabecular bone from femoral metaphyses (C) and vertebrae (D). Scale bar, 200 μm. Additional representative 3D μCT models of cortical bone are presented in Fig. S2A. *n* = 14 to 15 mice per group. Statistics used included a two-way analysis of variance with the Holm-Sidak posttest. *, *P* < 0.05 versus mock controls within bone and measurement type; #, *P* < 0.05 for long bones versus vertebrae.

To determine if B. burgdorferi bone colonization induced pathology, we measured areal and volumetric bone mineral density (aBMD and vBMD) in the left femora and L5 vertebrae from mock-infected and infected animals, using dual-energy X-ray absorptiometry (DXA) and microcomputed tomography (μCT). The effects of B. burgdorferi infection on trabecular and cortical bone were also compared using three-dimensional (3D) microarchitectural reconstructions from μCT. To distinguish between potential effects on trabecular and cortical bone, μCT analysis of cortical bone was performed in femora at the midshaft (composed mainly of cortical bone), whereas analysis of trabecular bone in femora was conducted at the distal metaphyses, where trabecular bone is more abundant (36). BMD values are expressed as percentages of the values for corresponding mock-infected animals (Fig. 3A).

To determine if changes in BMD in infected mice were clinically significant, we also calculated T-scores from the BMD values for vertebrae and femora. A T-score represents the number of standard deviations in the individual's BMD below the mean BMD of healthy young adults. T-scores 1 to 2.5 standard deviations lower than normal BMD are indicative of osteopenia, and T-scores more than 2.5 standard deviations lower than normal BMD correspond to osteoporosis (32). Means of individual T-scores were compared between B. burgdorferi-infected and mock-infected mice (Fig. 3B).

These experiments revealed that BMD of femoral trabecular bone was significantly lower in infected than in mock-infected mice (Fig. 3A). Trabecular BMD in vertebrae of infected animals was reduced but not significantly, and cortical BMD was not affected by B. burgdorferi infection (Fig. 3A). Examination of mean T-scores for bones from infected mice indicated that significant osteopenia was present in femora but not vertebrae (Fig. 3B). Representative reconstructed 3D models also showed visibly reduced bone volume and compactness from B. burgdorferi infection in the trabecular bone of femora, but the difference appeared less prominent in vertebral trabecular bone (Fig. 3C and D). Finally, to assess whether reductions in the BMD and T-scores were a function of increasing bacterial DNA burden, we calculated correlation coefficients for *flaB*:nidogen copy number ratios and areal and volumetric BMDs and T-scores in long bones and vertebrae (Table 1). All parameters were significantly negatively correlated with bacterial DNA burden, i.e., BMD and T-scores decreased as *flaB:nidogen* copy numbers increased. We concluded that B. burgdorferi infection was associated with clinically significant bacterial DNA load-dependent osteopenia in long bones but not vertebrae and that bone loss was confined to trabecular bone.

**TABLE 1:** Correlation analyses of relationships between B. burgdorferi DNA burden and DXA and μCT parameters

Bone type and correlation statistics	Correlation^*a*^ between bacterial DNA burden and:								
	aBMD	vBMD	T-score (aBMD)	T-score (vBMD)	BV/TV (μCT)	BS/BV (μCT)	Tb.N (μCT)	Tb.Pf (μCT)	Tb.Sp (μCT)
Long bones									
*r* (*P* value)	−0.50 (<0.05*)	−0.66 (<0.01*)	−0.51 (<0.01*)	−0.66 (<0.01*)	−0.66 (<0.01*)	0.61 (<0.01*)	−0.57 (<0.01*)	0.68 (<0.01*)	0.48 (<0.05*)
Vertebrae									
*r* (*P* value)	−0.12 (>0.05)	−0.079 (>0.05)	−0.049 (>0.05)	−0.079 (>0.05)	−0.13 (>0.05)	−0.018 (>0.05)	−0.15 (>0.05)	−0.019 (>0.05)	0.38 (>0.05)

a Asterisks indicate statistically significant correlations. Correlation analyses were performed by comparing DXA and μCT parameters to the mock background-corrected *flaB:nido* ratio for the same sample. Abbreviations: aBMD, areal bone mineral density; vBMD, volumetric bone mineral density; BV/TV, bone volume/tissue volume; BS/BV, bone surface/bone volume; Tb.N, trabecular number; Tb.Pf, trabecular pattern factor; Tb.Sp, trabecular separation.

### Effects of B. burgdorferi infection on trabecular and cortical microarchitecture in long bones and vertebrae.

To assess if trabecular bone microarchitecture deterioration was caused by infection, we measured the following parameters: BV/TV (the fraction of bone volume [BV] within total measured tissue volume [TV] of interest); BS/BV (the ratio of the bone surface [BS] to total bone volume [BV]), trabecular number (Tb.N, the average number of trabeculae per millimeter), trabecular separation (Tb.Sp, the mean distance between trabeculae), and the trabecular pattern factor (Tb.Pf, the intertrabecular connectivity, determined by assessing the relationship between convex and concave trabecular surfaces) (33). BV/TV, BS/BV, Tb.N, Tb.Sp, and Tb.Pf values were determined in three dimensions for the left femora and L5 vertebrae by using μCT. The same parameters, except Tb.Pf, were also measured in two dimensions in the proximal region of left tibiae and L3 vertebrae by histomorphometry performed in trichrome-stained bone sections. This permitted evaluation of bone structure by multiple methods, as well as comparison of effects of infection in different long bones.

**FIG 4:**
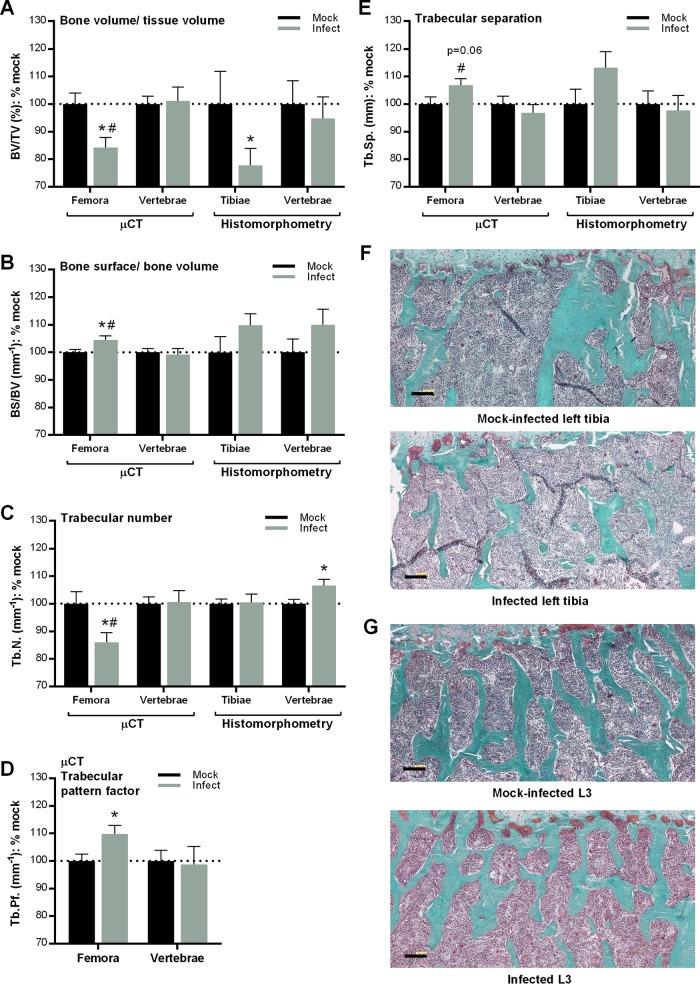
Effect of B. burgdorferi infection on trabecular bone microarchitecture. (A to E) Microarchitectural parameters of trabecular bone, measured by μCT (distal femoral metaphyses and L5 vertebrae) and histomorphometric analysis of trichrome-stained sections (proximal tibial metaphyses and L3 vertebrae). Shown are mean (± standard error of the mean) values normalized to means for mock-infected mice. Primary data are provided in Tables S1 and S2 in the supplemental material. *n* = 14 to 15 mice per group. Statistics used included a two-way analysis of variance with the Holm-Sidak posttest. *, *P* < 0.05 versus mock controls, within bone and measurement type; #, *P* < 0.05 for long bones versus vertebrae. (F and G) Representative sections from trichrome-stained left tibiae (F) and L3 vertebrae (G). Bar, 100 μm. Additional representative TRAP-stained sections are presented in Fig. S2B.

μCT analyses of trabecular bone from distal metaphyses of femora showed that bone comprised a significantly smaller proportion of total measured tissue volume in infected mice than in mock-infected controls (Fig. 4A, BV/TV), and BV/TV ratios decreased as B. burgdorferi DNA burden increased (Table 1). Infection was also associated with significantly increased bone surface per bone volume ratios in the femora (Fig. 4B, BS/BV), which also increased with increasing bacterial DNA load (Table 1). Trabecular number (Tb.N) was significantly lower in femora of infected animals (Fig. 4C) and was negatively correlated with B. burgdorferi DNA burden (Table 1). Moreover, the trabecular pattern factor (Tb.Pf) parameter was greater in infected femora (Fig. 4D) and increased with bacterial DNA load (Table 1), indicating more poorly connected spongy lattices (33). A corresponding increase in trabecular separation (Tb.Sp) that was positively correlated with *flaB* copy number was observed in infected femora (Table 1; Fig. 4E). Histomorphometry analyses of trabecular bone from trichrome-stained sections of proximal tibiae also revealed significant reductions in BV/TV in infected mice (Fig. 4A). BS/BV (Fig. 4B) and Tb.Sp (Fig. 4E) values increased by a similar percentage in femora (μCT) and tibiae (histomorphometry) of infected animals, but the changes observed in tibiae were not significant, possibly due to inherently greater variations in histomorphometry sectioning and measurements. Tb.N did not differ in tibiae and femora of mock-infected and infected mice (Fig. 4C). Collectively, these analyses indicated that trabecular bone in the long bones of B. burgdorferi-infected mice was more porous and was composed of smaller, more poorly connected trabeculae, consistent with the observed osteopenia.

In contrast to the findings with long bones, neither μCT nor histomorphometry nor *flaB* copy number correlation analyses of vertebrae revealed significant infection-dependent changes in BV/TV, BS/BV, Tb.Sp, or Tb.Pf (Fig. 4A, B, and D; Table 1). Representative sections also showed that bone volume was reduced more dramatically by infection in tibiae than in vertebrae (Fig. 4F and G). Additionally, Tb.N was similar in μCT-analyzed vertebrae from both experimental groups but was significantly greater in the vertebrae of infected than mock-infected mice when analysis was performed by histomorphometry (Fig. 4C). The discrepancy in these results suggests that the effect of infection on trabecular number was likely mild and was possibly less frequently observed in some vertebrae than others, likely due to a low B. burgdorferi DNA load in the vertebrae compared (Fig. 2). Together, these data showed that B. burgdorferi infection did not affect vertebral trabecular bone microarchitecture as strongly as it affected long bone microarchitecture, consistent with the results of BMD assays.

To assess the effects of B. burgdorferi infection on cortical bone microarchitecture, we measured cortical bone perimeter (B.Pm), thickness, cross-sectional area, and anteroposterior (AP) and mediolateral (ML) diameters in femoral midshafts via μCT. None of these properties differed significantly in infected versus mock-infected groups (see Table S1 in the supplemental material). Furthermore, three-point bending at the midshafts of the same femora revealed that infection did not affect cortical bone biomechanical properties (Table 2). Together with the results of BMD assays, these results implied that B. burgdorferi infection did not affect the structure or function of cortical bone.

**TABLE 2:** Biomechanical properties of femoral midshafts, assessed by three-point bending

Biomechanical category and property	Value (mean ± SEM) for group^*a*^	
	Mock infected	Infected
Structural properties		
Peak load (N)	21.78 ± 0.68	22.15 ± 0.34
Failure displacement (mm)	0.13 ± 0.0036	0.14 ± 0.0031
Work to failure (mJ)	1.48 ± 0.077	1.58 ± 0.053
Stiffness (N/mm)	198.50 ± 4.72	193.10 ± 3.40
Mechanical properties		
Ultimate stress (MPa)	183.40 ± 5.23	191.20 ± 7.38
Failure strain (%)	1.94 ± 0.064	1.99 ± 0.056
Toughness (mJ/mm^3^)	168.70 ± 10.56	166.50 ± 8.07
Young's modulus (GPa)	11.16 ± 0.17	11.41 ± 0.23

a No significant differences were found between the mock and infected groups for any measured parameter (two-tailed unpaired *t* test; *P* > 0.05).

Collectively, these data indicated that B. burgdorferi infection significantly disrupted the trabecular microarchitecture of long bones without affecting the vertebrae or cortical bone.

### Bone loss in long bones of B. burgdorferi-infected mice is associated with reduced numbers of osteoblasts but does not affect osteoblast activity or osteoclast numbers.

Healthy bone remodeling in response to stressors results from balance between bone-degrading activities of osteoclasts and the bone-building activities of osteoblasts (34). Increased osteoclastogenesis and osteoclast activity can result in increased number and size of osteoclasts at the bone surface, which lead to net bone resorption. This is frequently observed in bone loss due to local or systemic inflammation (8). In contrast, osteoblast apoptosis (i.e., decreased osteoblast numbers) and inhibition of osteoblast function (i.e., lower osteoid production, slower mineralization rate) are more prominent in bone loss associated with direct infection of bone (i.e., osteomyelitis) (9).

**FIG 5:**
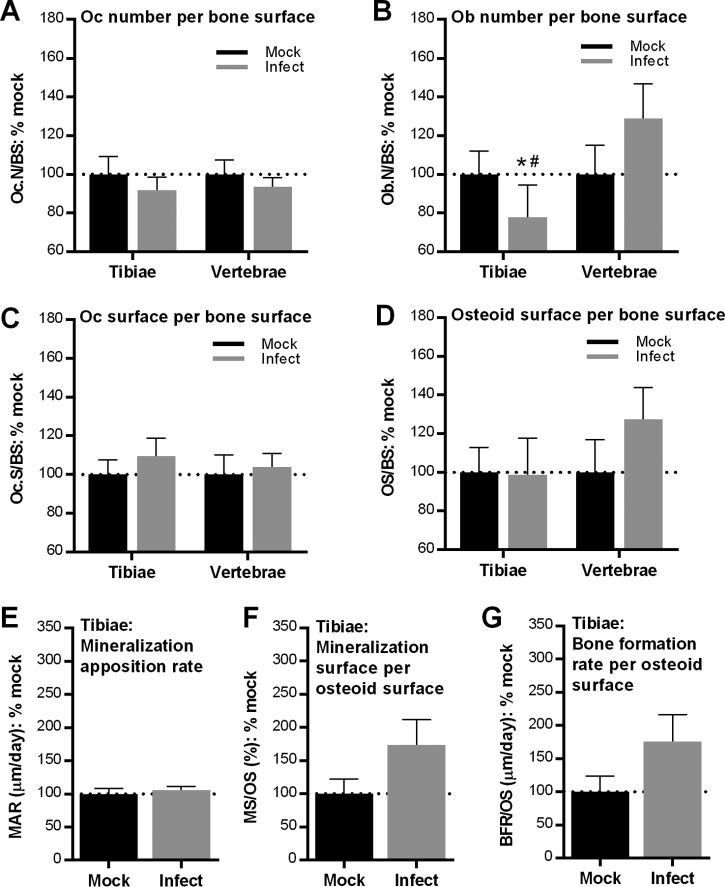
Effect of B. burgdorferi infection on cellular composition and activity in bone (A to D). Static histomorphometry analysis of numbers of osteoclasts (Oc) and osteoblasts (Ob) per bone surface (A and B) and osteoclast and osteoid surface per bone surface (C and D). Osteoclast parameters were measured in TRAP-stained sections of right proximal tibial metaphyses and L4 vertebrae. Osteoblast and osteoid parameters were measured in trichrome-stained sections of left proximal tibial metaphyses and L3 vertebrae. (E and G) Dynamic histomorphometry analysis of mineral apposition rate/day (E), mineralization surface/osteoid surface (F), and bone formation rate per osteoid surface (G) in left proximal tibial metaphyses, measured by imaging with fluorescent calcein green. For all groups, mean (± standard error of the mean) values for parameters were normalized to the mean value for mock-infected mice. Primary data are provided in Table S2 in the supplemental material. Statistics used included a two-way analysis of variance with the Holm-Sidak posttest (A and B) and two-tailed unpaired *t* tests (C to E). *, *P* < 0.05 versus mock controls within bone and measurement type; #, *P* < 0.05 for long bones versus vertebrae.

To determine whether osteopenia in trabecular regions of long bones in B. burgdorferi-infected mice was associated with changes in bone cell numbers, we measured the osteoclast number per bone surface (Oc.N/BS) in tartrate-resistant acid phosphatase (TRAP)-stained sections of right proximal tibial metaphyses and L4 vertebrae, and also osteoblast numbers per bone surface (Ob.N/BS) in trichrome-stained sections of the left proximal tibial metaphyses and L3 vertebrae (Fig. 5A and B). We also measured the ratio of total osteoclast surface to total bone surface (Oc.S/BS) and osteoid surface to bone surface (OS/BS) from the same sections (Fig. 5C and D).

Since B. burgdorferi-induced pathology in most tissues is primarily due to host inflammatory responses to these bacteria (3), we expected that bone loss in infected mice would be accompanied by significant osteoclast-driven bone resorption. However, we found that osteoclast numbers (Fig. 5A) and coverage of bone surfaces (Fig. 5C) did not significantly increase in either the tibiae or vertebrae of infected mice. Instead, we observed that bone loss was accompanied by reduced osteoblast numbers at bone surfaces in tibiae (Fig. 5B), although reductions in osteoblast numbers did not appear to depend on bacterial DNA burden (Table S3). Osteoblast numbers in vertebrae were significantly greater than in tibiae (Fig. 5B), suggesting that differences in osteoblast numbers possibly contributed to differences in the bone loss phenotypes in these bones.

Despite reduced osteoblast numbers in the tibiae (Fig. 5B), osteoid production in bones of infected mice was not impaired (Fig. 5D). This implied that infection did not inhibit the activity of the osteoblasts present in bone. To further test this conclusion, rates of mineralized bone formation over time were examined via dynamic histomorphometry in calcein-labeled bone (Fig. 5E to G). Mineralization apposition rates (MARs) in infected and mock-infected animals were similar (Fig. 5E), as was the ratio of mineralization surface area to osteoid surface area (MS/OS) (Fig. 5F). Furthermore, infection did not reduce bone formation rate/osteoid surface ratios (BFR/OS) (Fig. 5G), implying that growth rates for mineralized bone were not affected when adjusted for osteoid volume. Thus, we concluded that although osteoblast numbers were reduced, osteoid production and mineralization were not affected by infection. Collectively, these data indicated that B. burgdorferi infection-dependent bone loss was associated with reductions in osteoblast numbers in long bones, but not with increased osteoclastogenesis, increased osteoclast activity, or impaired osteoblast activity.

## DISCUSSION

In this study, we found that B. burgdorferi infection in mice is associated with infectious dose- and time-dependent reductions in ALP, a serum biomarker of bone formation in mice. Infection is also associated with B. burgdorferi DNA burden-dependent osteopenia and disrupted trabecular microarchitecture in long bones but not in vertebrae. These effects were not associated with increased osteoclast-dependent bone resorption, inhibition of osteoblast synthesis of osteoid cells, or bone mineralization. Instead, trabecular bone loss was associated with osteoblast number reductions in long bones but not in vertebrae. It is likely that differences in the effect of B. burgdorferi infection in long bones and vertebrae were due at least in part to differences in bacterial DNA burdens in these bones. Many bone loss parameters were significantly correlated with *flaB* copy number in long bones but not in vertebrae, suggesting that B. burgdorferi did not appear to colonize and/or persist as effectively in vertebral bone. However, other differences between long bones and vertebrae may have also contributed to differences in susceptibility to B. burgdorferi infection and infection outcomes, since long bones and vertebrae have been observed to be affected differently by systemic factors (e.g., rosiglitozone treatment) for reasons that remain unknown (30).

Although bone pain and bone involvement have been described in patients with Lyme disease (5, 12–18, 20, 21) and B. burgdorferi has been detected in human bone and bone marrow (10–13), the possibility that B. burgdorferi may infect and cause pathology in bone at sites other than articular surfaces has not been widely investigated. We found that B. burgdorferi infection in mice causes a level of osteopenia in the trabecular regions of long bones that would be considered a clinically significant finding in humans. However, structural and biomechanical properties of cortical bone were not affected by infection. It will be important to determine if conditions such as prolonged B. burgdorferi infection or infection in the context of preexisting osteopenia or osteoporosis would eventually result in a clinically symptomatic phenotype in cortical bone. It may also be useful to determine if B. burgdorferi infection in humans is associated with increased rates and severity of osteopenia and osteoporosis, and/or increased risk of fracture outcomes.

Bone loss results from an imbalance between the bone-resorbing activities of osteoclasts and bone-building functions of osteoblasts. Our data suggest that bone loss at 4 weeks postinfection was not primarily due to increased osteoclastogenesis or osteoclast activation, although it is possible that osteoclast-driven bone loss occurred earlier in infection. This is plausible, because tumor necrosis factor alpha (TNF-α), interleukin-1 (IL-1), IL-6, and IL-17, which are systemically upregulated in response to B. burgdorferi infection (35–38), are some of the major inflammatory cytokines that stimulate osteoclastogenesis and osteoclast activation (8, 39). TNF-α, IL-1, and IL-6 also suppress differentiation of osteoblasts (8), which is consistent with our observation that osteoblast numbers were reduced in bones which exhibited osteopenia. However, regardless of whether increased osteoclast-dependent resorption was responsible for early bone loss in infection, data from the present study suggest that osteoblast deficiency makes important, long-lasting contributions to bone loss.

The major causes of osteoblast-related abnormalities in bone disease are loss or reduction of signals required for osteoblast differentiation (e.g., hormones or local regulatory proteins, such as bone morphogenetic proteins), differentiation of bone marrow mesenchymal stem cells into cells of nonosteoblastic lineage, premature osteoblast cell death, and inhibition of osteoblast activities (i.e., osteoid synthesis and mineralization) (40). We did not find that osteoid production or mineralization by the osteoblasts present in bones of infected mice were impaired, implying that osteoblast abnormalities occurred instead due to suppression of osteoblastogenesis or reduced osteoblast viability. It is unknown if B. burgdorferi infection affects mesenchymal stem cell differentiation or other major regulators of osteoblastogenesis, such as growth hormones, growth factors, estrogen, and parathyroid hormone (40). However, one of B. burgdorferi's complement-regulating surface proteins, CRASP-1, is known to bind bone morphogenetic protein-2 (BMP-2) (41), a potent inducer of osteoblast differentiation (40). Treatment with vitamin D, another key regulator of osteoblastogenesis, has also been shown to reduce the severity of Lyme arthritis in mouse models (42). Additionally, direct or indirect bacterial induction of cell death in osteoblast lineage cells is a known important mediator of bone loss seen in osteomyelitis caused by other bacterial species (9). B. burgdorferi is known to induce cell death in a number of cell types, including glia, neurons, oligodendrocytes, lymphocytes, monocytes, and macrophages (43–48), but the effect of this bacterium on osteoblast biology and cell death have not yet been examined. These literature observations, together with our findings, warrant future investigations on the effect of B. burgdorferi infection on osteoblastogenesis and osteoblast viability.

In summary, this paper presents the first animal study directly demonstrating an effect of B. burgdorferi infection on bone tissue deterioration at sites other than articular surfaces involved in Lyme arthritis. Since these observations may have implications for our understanding of Lyme disease in humans, further investigation of the mechanisms underlying bone loss in response to B. burgdorferi infection is warranted.

## MATERIALS AND METHODS

### Ethics statements.

All animal procedures were approved by the University of Toronto Animal Care Committee (protocol 20011501). Mice were housed in a biosafety level 2 room in groups of 2 to 4 per cage, with *ad libitum* access to food and water. Mice were anesthetized to a surgical plane of anesthesia by intraperitoneal injection of 200 mg/kg of body weight ketamine hydrochloride (Rogar/STB, Montréal, QC, Canada) and 10 mg/kg xylazine (MTC Pharmaceuticals, Cambridge, ON, Canada) before tissue harvesting and euthanasia.

### Animal husbandry and dietary conditions.

Upon arrival, 4-week-old male C3H/HeNCrl mice (Charles River, Montréal, QC, Canada) were fed standard chow (Teklad 2018 rodent chow; Harlan Laboratories, Mississauga, ON, Canada). At 12 weeks of age, mice were switched to a maintenance diet containing 0.6% calcium (Teklad 2014 rodent chow; Harlan Laboratories) until sacrifice.

### B. burgdorferi cultivation and mouse infections.

The B. burgdorferi strain used in this study was B31 5A4-derived GCB726 (49). As described elsewhere (50), this strain was cultivated to log phase in Barbour-Stoenner-Kelly II (BSK-II) medium before infection experiments. At 12 weeks of age, randomly assigned mice were inoculated subcutaneously at the dorsal lumbar midline with B. burgdorferi or BSK-II medium alone (mock infection). With the exception of the experiments presented in Fig. 1 and also Fig. S1 in the supplemental material, mice were infected with 10^4^ B. burgdorferi for a period of 4 weeks. Each of these mice was also intraperitoneally injected with freshly prepared, 30 mg/kg 0.6% calcein green (Sigma-Aldrich Canada, Oakville, ON) at 9 and 2 days prior to sacrifice, to label newly formed bone.

### Harvesting and preparation of sera and bones.

Blood was collected by cardiac puncture from anesthetized mice before euthanasia by cervical dislocation, as described previously (50). Serum was harvested after overnight incubation at 4°C by centrifugation at 10,000 × *g* for 10 min.

The following bones were collected for all other analyses in this study: femora, tibiae, and L3 to L6 vertebrae. The left femora and L5 vertebral columns were cleaned of adherent soft tissue, wrapped in saline-soaked gauze, and stored at −20°C. These bones were thawed overnight at 4°C before DXA, μCT, and biomechanical analyses. Tibiae were excised and clipped at the midshaft into proximal and distal halves. Proximal metaphyses of the right tibiae and L4 vertebral columns were fixed immediately in 10% buffered formalin (Sigma) and decalcified in 14% EDTA (pH 7.4) at 4°C for 21 days, with solutions replaced every 2 days. These samples were then processed by the Toronto Centre for Phenogenomics (see the “Bone histomorphometry” section). Proximal metaphyses of the left tibiae and L3 vertebral columns were fixed in 70% ethanol and stored at 4°C until submission to the Toronto Centre for Phenogenomics for processing (see details in the “Bone histomorphometry” section). Distal tibial halves and L6 vertebral bodies cleaned of adherent soft tissue were stored at −80°C until DNA extraction for qPCR analysis. All extractions were performed using bone distal from joints to ensure that any bacterial DNA detected was not joint derived.

### Serum ALP activity assays.

Serum ALP activity was measured in technical duplicates using a colorimetric ALP assay kit (Abcam, Cambridge, UK) following the manufacturer's instructions.

### DNA extraction and qPCR measurement of B. burgdorferi DNA copy number.

For DNA extraction, bones were thoroughly cleaned of peripheral soft tissue, and bone marrow was removed by flushing with a syringe. Cleaned, rinsed bones were then pulverized with a mortar and pestle on dry ice. DNA was extracted from pulverized bone using Qiagen DNeasy tissue extraction kits (Toronto, ON, Canada) as per the manufacturer's instruction. Concentration of extracted DNA were measured using a Nanodrop spectrophotometer (Thermo Fisher Scientific, Waltham, MA, USA). Measurement of numbers of copies of B. burgdorferi flaB DNA by qPCR was performed in technical sextuplicates as described previously (50), using triplicate logarithmic dilutions (10^1^ to 10^6^ copies) of plasmid pTM222 (which contained a copy of the *flaB* DNA sequence) as a standard curve. The mean of technical replicates was used for subsequent analyses, and findings were expressed as the ratio per 1,000 copies of mouse nidogen DNA measured by qPCR from the same sample. For each bone type, the median *flaB:nidogen* ratio from mock-infected mice was subtracted from *flaB:nidogen* ratios from infected and mock-infected animals.

qPCR for mouse nidogen was performed with logarithmic dilutions of plasmid standard pTM221 containing the nidogen target amplification sequence (standard range, 1 to 10^8^ copies/reaction mixture). pTM221 (Escherichia coli strain GCE1844) was cloned by PCR amplifying the nidogen target sequence from mouse genomic DNA with primers nido.F (5′-CGAGCCACAGAATACCATCC-3′) and nido.R (5′-GGACATACTCTGCTGCCATC-3′) into the pJET1.2/Blunt cloning vector using the CloneJet PCR cloning kit (Thermo-Fisher Scientific), followed by sequencing of the insert. qPCR was performed as described for *flaB* (50), using primers nido.F and nido.R (51). qPCR conditions were 95°C for 5 min, followed by 50 cycles of 98°C for 5.75 s, 60°C for 2.75 s, and 86°C for 5 s, and then melt curve analysis ranging from 60°C to 95°C. Average nidogen copy numbers obtained from triplicate reaction mixtures were used for normalization of *flaB* copy numbers.

### BMD and morphometry measurements.

Measurement of areal bone mineral density (aBMD, in grams per centimeter squared) in left femora and L5 vertebral bodies was performed by DXA using a PIXImus mouse bone densitometer (GE Medical Systems, Madison, WI). Measurements of volumetric bone mineral density (vBMD, in grams per cubic centimeter) and morphometry from trabecular and cortical bones of left femora, as well as from L5 vertebral bodies, were performed after DXA analysis by μCT, using a SkyScan 1174 μCT scanner (Bruker microCT, Kontich, Belgium). Images were acquired at 50 kV and 800 μA, and a 0.25-mm aluminum filter was applied for noise removal. Two hydroxyapatite (HA) phantoms were scanned and used to calculate vBMD. Structural indices were computed using the SkyScan CT Analyzer software. Following image acquisition, three-dimensional reconstruction was performed to calculate bone microarchitecture parameters. Morphometry measurements were performed with global thresholding to exclude soft tissue (trabecular bone, 0.9 g HA/cm^3^; gray value, 65; cortical bone, 1.1 g HA/cm^3^; gray value, 100).

For trabecular bone analyses, distal femoral metaphyses and L5 vertebrae were scanned at a voxel size of 6.1 μm^3^. The volume of interest (VOI) for femora consisted of a 400-slice (2.4-mm) section extending 50 slices (0.3 mm) from the first cartilage bridge of femur metaphyses. The VOI for L5 was between the growth plates, with a 50-slice (0.3-mm) offset from each. For cortical bone analyses, femoral middiaphyses were scanned at 11.6 μm^3^. The VOI for cortical bone was a 100-slice (1.16-mm) section at the middiaphysis.

### Biomechanical testing.

After μCT analysis, biomechanical properties of the left femorae were assessed by three-point bending at the midshaft, using an ElectroPuls E1000 machine (Instron Corp., Canton, MA, USA) with a 100-N load cell. Femora were placed anteriorly face up on two supports, 7 mm apart. A pretest of 10 cycles (maximum, 5-N load; minimum, 2-N load) was applied to each femur. A destructive load was then vertically applied to the midshaft at 2.0 mm/min until failure. Load deformation curves derived from biomechanical testing were generated using the accompanying Bluehill 3 software (Instron). Parameters were calculated using geometry measurements for cortical bone from the μCT data.

### Bone histomorphometry.

Tartrate-resistant acid phosphatase (TRAP) staining for identification of osteoclasts was performed on 5-μm-thick sections cut from formalin-fixed, decalcified right tibiae and L4 vertebrae embedded in paraffin. Ethanol-fixed, undecalcified left tibiae and L3 vertebrae were embedded in methylmethacrylate, sectioned, and stained with Goldner's trichrome (4-μm-thick sections) or left unstained for measurement of calcein fluorescence (7-μm-thick sections). All histology services were performed by the Toronto Phenogenomics Centre.

Static and dynamic histomorphometry analyses of TRAP-stained, trichrome-stained, and calcein green-labeled sections were performed manually using BioQuantOsteo v11.2.6. All histomorphometric measurements were performed and analyzed following guidelines from the American Society of Bone and Mineral Research (52).

### Statistical analyses.

Statistical analyses were performed in Prism v.6.0 (GraphPad Software, La Jolla, CA, USA). Specific tests used for analyses are indicated in the figure legends and table footnotes. Pearson correlation analyses were performed for log-transformed *flaB* copy numbers per 1,000 nidogen copies and various bone parameters from each mouse. Data were considered statistically significant at a confidence level of 95% (*P* < 0.05).

## Supplementary Material

Supplemental material
