# Supplementary material for: The Lyme Disease Pathogen Borrelia burgdorferi Infects Murine Bone and Induces Trabecular Bone Loss
Source: Infect Immun. 2017 Jan 26;85(2):e00781-16. doi: 10.1128/IAI.00781-16 (PMC5278181; doi:10.1128/IAI.00781-16)
Supplement: Supplemental material [file supp_85_2_e00781-16__index.html]

The Lyme Disease Pathogen Borrelia burgdorferi Infects Murine Bone and Induces Trabecular Bone Loss — Supplemental material 

# The Lyme Disease Pathogen Borrelia burgdorferi Infects Murine Bone and Induces Trabecular Bone Loss

## Supplemental material

- Supplemental file 1 -

  Fig. S1. Effect of *B. burgdorferi* infection on serum ALP: primary data. Fig. S2. Representative images: 3-D models of cortical bone and TRAP-stained trabecular bone sections. Table S1. Femoral and vertebral microarchitectural properties measured by DXA and μCT. Table S2. Histomorphometry measurements: tibiae and vertebrae. Table S3. Correlation analysis of relationship between *B. burgdorferi* DNA burden and osteoblast and osteoclast numbers and cell surface: bone surface ratios.

  PDF, 1.4M
