# Supplementary material for: The Lyme Disease Pathogen Borrelia burgdorferi Infects Murine Bone and Induces Trabecular Bone Loss
Source: Infect Immun. 2017 Jan 26;85(2):e00781-16. doi: 10.1128/IAI.00781-16 (PMC5278181; doi:10.1128/IAI.00781-16)
Supplement: Supplemental material [file IAI.00781-16_zii999091967s1.pdf]

SUPPLEMENTAL INFORMATION

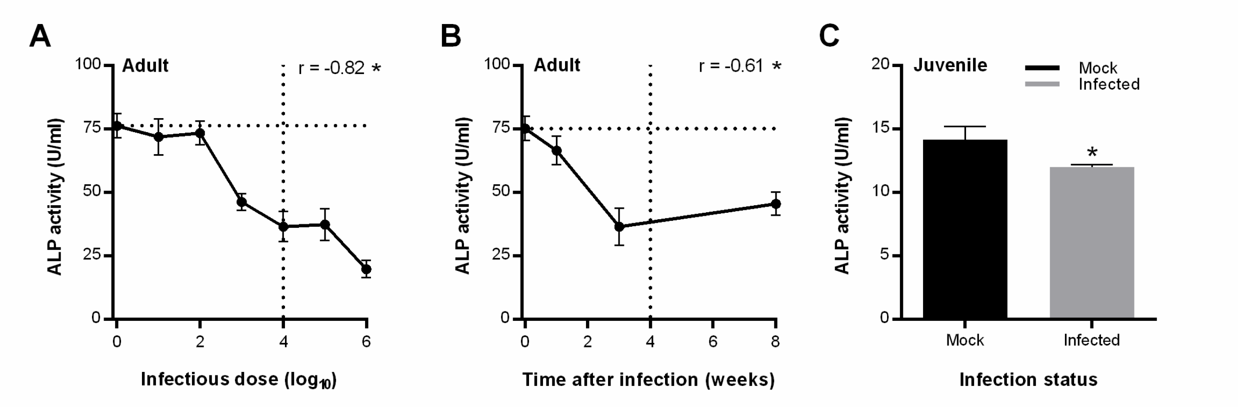

Figure S1: Effect of *B. burgdorferi* infection on serum ALP: Primary data

Mean ± SEM serum alkaline phosphatase (ALP) activity. **(A-B)** Serum ALP activity in adult (16-17 week old) male C3H/HeN mice infected for 3 weeks with 10^1-10^6 B31-derived GCB726 *B. burgdorferi* (A) or for 1, 3 and 8 weeks with 10^4 bacteria (B). N=5 mice/group (A), 10 mice/group (B). Statistics: Pearson correlation analysis (r). * indicates $p<0.05$ vs dose (A) and time (B). **(C)** Serum ALP activity in juvenile (3-4 week old) male C3H/HeN mice infected for 4 weeks with 10^4 *B. burgdorferi*. Mock: age-matched mice inoculated with vehicle alone. N=10 mice/group. Statistics: two-tailed unpaired *t* test. * indicates $p<0.05$ vs mock.

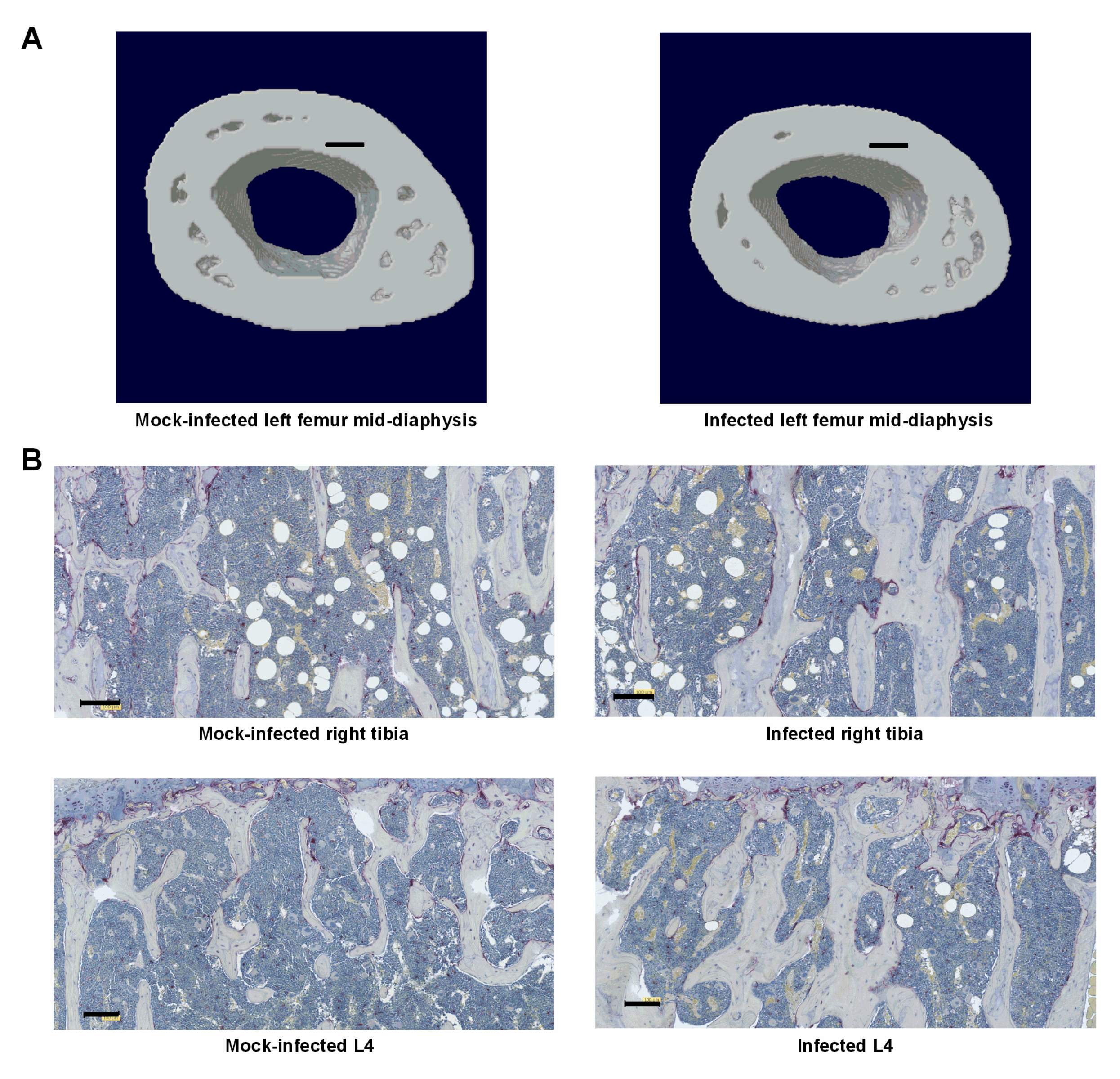

Figure S2: Representative images: 3D models of cortical bone and TRAP-stained trabecular bone sections

(A) 3D models of cortical bone at femoral mid-diaphyses, obtained by µCT. Scale bar and ticks: 200 µm. **(B)** TRAP-stained right tibiae and L4 vertebrae. Scale bar: 100 µm.

Table S1: Femoral and vertebral microarchitectural properties measured by DXA and µCT

	Mock-infected	Infected
	Trabecular bone	
	Left femora	
Areal BMD (g/cm^2)	0.069 ± 0.00068	0.065 ± 0.0071 *
Volumetric BMD (g/cm^3)	0.33 ± 0.0058	0.3086 ± 0.0073 *
Bone volume/tissue volume (%)	13.05 ± 0.52	10.99 ± 0.47 *
Bone surface/bone volume (mm^{-1})	62.93 ± 0.066	65.75 ± 0.95 *
Trabecular number (mm^{-1})	2.02 ± 0.089	1.74 ± 0.072 *
Trabecular pattern factor (mm^{-1})	21.35 ± 0.52	23.44 ± 0.65 *
Trabecular separation (mm)	0.22 ± 0.0060	0.24 ± 0.0051 (p=0.06)
	L5 vertebrae	
Areal BMD (g/cm^2)	0.030 ± 0.00096	0.028 ± 0.0011 (p=0.07)
Volumetric BMD (g/cm^3)	0.29 ± 0.0074	0.27 ± 0.011
Bone volume/tissue volume (%)	14.87 ± 0.42	15.04 ± 0.74
Bone surface/bone volume (mm^{-1})	2.50 ± 0.063	2.51 ± 0.10
Trabecular number (mm^{-1})	15.83 ± 0.62	15.62 ± 1.03
Trabecular pattern factor (mm^{-1})	64.37 ± 0.88	63.87 ± 1.44
Trabecular separation (mm)	0.29 ± 0.0083	0.28 ± 0.0089
	Cortical bone: *Left femora*	
Volumetric BMD (g/cm^3)	1.34 ± 0.0093	1.34 ± 0.020
Periosteal perimeter (mm)	8.21 ± 0.12	8.11 ± 0.10
Cortical thickness (mm)	0.33 ± 0.0037	0.33 ± 0.0052
Cross-sectional bone area (mm^2)	1.09 ± 0.014	1.08 ± 0.020
Anteroposterior (AP) diameter (mm)	1.18 ± 0.012	1.20 ± 0.012
Mediolateral (ML) diameter (mm)	1.75 ± 0.019	1.72 ± 0.016
Closed porosity (%)	0.60 ± 0.20	0.44 ± 0.093

Mean ± SEM values for all parameters. All parameters except areal BMD were measured by µCT. Statistics: two-tailed unpaired *t* test. *: $p<0.05$ vs mock.

Table S2: Histomorphometry measurements: tibiae and vertebrae

	**Mock-infected**	**Infected**
Static histomorphometry: TRAP-stained slides		
Right tibiae		
Bone volume/tissue volume (%)	26.47 ± 1.51	29.55 ± 1.51
Bone surface/bone volume (mm^{-1})	29.39 ± 1.94	26.50 ± 1.11
Osteoclast number/bone surface (mm^{-1})	5.19 ± 0.57	4.63 ± 0.30
Osteoclast surface/bone surface (%)	8.00 ± 0.58	9.11 ± 0.76
L4 vertebrae		
Bone volume/tissue volume (%)	27.93 ± 1.77	27.73 ± 2.36
Bone surface/bone volume (mm^{-1})	35.77 ± 1.64	37.85 ± 3.05
Osteoclast number/bone surface (mm^{-1})	4.27 ± 0.32	4.17 ± 0.22
Osteoclast surface/bone surface (%)	7.14 ± 0.73	7.92 ± 0.54
Static histomorphometry: Trichrome-stained slides		
Left tibiae		
Bone volume/tissue volume (%)	20.56 ± 2.71	14.91 ± 1.24 *
Bone surface/bone volume (mm^{-1})	39.67 ± 2.66	45.12 ± 1.76 (p=0.09)
Trabecular number (mm^{-1})	4.73 ± 0.086	4.79 ± 0.15
Trabecular separation (mm)	0.11 ± 0.0058	0.12 ± 0.0061
Osteoblast number/bone surface (mm^{-1})	0.91 ± 0.12	0.72 ± 0.17 *
Osteoid volume/bone volume (%)	1.89 ± 0.30	2.03 ± 0.45
L3 vertebrae		
Bone volume/tissue volume (%)	23.95 ± 2.02	22.70 ± 1.87
Bone surface/bone volume (mm^{-1})	40.52 ± 1.96	44.56 ± 2.31
Trabecular number (mm^{-1})	5.41 ± 0.086	5.77 ± 0.12 *
Trabecular separation (mm)	0.084± 0.0038	0.082 ± 0.0045
Osteoblast number/bone surface (mm^{-1})	0.63 ± 0.094	0.81 ± 0.11
Osteoid volume/bone volume (%)	1.35 ± 0.24	1.77 ± 0.24
Dynamic histomorphometry: Calcein imaging		
Left tibiae		
Mineral apposition rate (mcm/day)	1.33 ± 0.11	1.41 ± 0.075
Mineralization surface/osteoid surface (%)	202.70 ± 45.16	351.40 ± 77.75 (p=0.13)
Bone formation rate/osteoid surface (mcm/day)	2.86 ± 0.67	5.03 ± 1.15 (p=0.14)
Mineralization surface/bone surface (%)	16.32 ± 0.82	19.54 ± 1.23 (p=0.051)
Bone formation rate/bone surface (mcm/day)	0.14 ± 0.012	0.17 ± 0.014 (p=0.076)

Mean ± SEM values for all parameters. Statistics: two-tailed unpaired *t* test. *: $p<0.05$ vs mock.

Table S3: Correlation analysis of relationship between *B. burgdorferi* DNA burden and osteoblast and osteoclast numbers and cell surface: bone surface ratios

Correlation between bacterial DNA burden & / Correlation parameters	Oc.N/BS	Ob.N/BS	Oc.S/BS	OS/BS
		Long bones		
r	-0.085	0.030	0.34	0.18
**p value**	>0.05	>0.05	>0.05	>0.05
		Vertebrae		
r	-0.17	0.21	-0.17	0.0056
**p value**	>0.05	>0.05	>0.05	>0.05

r: Pearson correlation coefficient. Oc.N/BS: osteoclast number/bone surface. Ob.N/BS: osteoblast number/bone surface. Oc.S/BS: osteoclast surface/bone surface. OS/BS: osteoid surface/bone surface. Correlation analyses were performed by comparing cell number and surface parameters to the mock background-corrected *flaB:nido* ratio for the same sample.
